# Diversity of Microorganisms and Their Metabolites in Food

**DOI:** 10.3390/microorganisms12010205

**Published:** 2024-01-19

**Authors:** João Miguel Rocha, Biljana Kovacevik, Sanja Kostadinović Veličkovska, Mercedes Tamame, José António Teixeira

**Affiliations:** 1 Universidade Católica Portuguesa, CBQF—Centro de Biotecnologia e Química Fina—Laboratório Associado, Escola Superior de Biotecnologia, Rua Diogo Botelho 1327, 4169-005 Porto, Portugal; 2 LEPABE—Laboratory for Process Engineering, Environment, Biotechnology and Energy, Faculty of Engineering, University of Porto (FEUP), Rua Dr. Roberto Frias, 4200-465 Porto, Portugal; 3 AliCE—Associate Laboratory in Chemical Engineering, Faculty of Engineering, University of Porto (FEUP), Rua Dr. Roberto Frias, 4200-465 Porto, Portugal; 4 Faculty of Agriculture, University “Goce Delčev”, Krste Misirkov bb, 2000 Štip, North Macedonia; biljana.kovacevik@ugd.edu.mk (B.K.); sanja.kostadinovik@ugd.edu.mk (S.K.V.); 5 Institute of Functional Biology and Genomics (IBFG), CSIC-University of Salamanca, 37007 Salamanca, Spain; tamame@usal.es; 6 Centre of Biological Engineering, University of Minho, 4710-057 Braga, Portugal; jateixeira@deb.uminho.pt

Throughout history as well as the present, food microorganisms have been proven to play a significant role in human life. Some of them appear to contaminate food during storage and cause spoilage. Foodborne microorganisms that do not cause any bad smell and have no taste or surface appearance on food, such as *Salmonella* spp., *Escherichia* spp., and *Listeria* spp., are of particular concern [1]. Because of these characteristics, these microorganisms are the most troublesome, and their presence in food often leads to foodborne outbreaks. To prevent their multiplication, chemical preservatives are usually included as an ingredient in food. Nonetheless, consumers’ demand for more natural and minimally processed food promotes the use of other, more naturally occurring, alternatives. Microorganisms whose metabolites possess antimicrobial properties have been utilized in natural food preservation and introduced to the food industry [2]. Lactic acid bacteria (LAB) are particularly important and used for this purpose in practice. Their properties are widely studied, and a great number of their metabolites have been identified to possess antimicrobial properties [3]. Most of them include some reuterins, bacteriocins, organic acids, amino acids, monohydroxy fatty acids, exopolysaccharides, etc. [4]. More precisely, reuterins are antimicrobial compounds produced by certain strains of *Limosilactobacillus reuteri*. *Limosilactobacillus reuteri* are heterofermentative lactic acid bacteria found in a variety of ecological niches like food fermentations, such as during sourdough and bread production. Reuterins can be found in hydrated, non-hydrated, and dimeric forms of 3-hydroxypropionaldehyde (3-HPA), and their free thiol groups are usually related with the antimicrobial potential of reuterins [5]. 

Bacteriocins are another wide and versatile group of bioactive bacterial peptides or proteins with high antimicrobial potential against other bacteria. Bacteriocins include peptides or proteins of variable biochemical properties, molecular weight, mechanism of action, spectrum of activity, location, and sequence of amino acids [6]. The term ‘bacteriocin’ denotes a toxic protein or peptide produced by any type of bacteria and is active on related bacteria, but it is not harmful for the producing cell. This term was inspired by the antimicrobial activity of colicin, the first bacteriocin described, which was produced by *Escherichia coli* [7]. Bacteriocins produced by Gram-positive bacteria are divided into four groups. The first group covers post-translationally modified bacteriocins containing unusual amino acids (lantibiotics); the second group includes heat, unmodified, and non-lanthionine-containing bacteriocins; the third group represents bacteriocins with molecular weights above 30 kDa; and lastly, the forth group covers bacteriocins containing lipid or carbohydrate moieties. Bacteriocins from Gram-negative bacteria are colicins and microcins. Bacteriocins manifest their antibacterial activity four different ways: bactericidal activity due to the formation of pores in the cell membrane, the inhibition of cell wall component biosynthesis, affecting the activity of autolytic enzymes, and inhibiting bacterial spores’ formation. The most important role of bacteriocins is that they can be used as natural preservatives in bread and other foodstuffs, with the aim of avoiding harmful and potentially cancerogenic synthetic additives [8]. 

In addition to this, LAB also have many other useful features [9]. A large number are naturally present in food and affect its rheological and sensory characteristics. The microorganism species that prevails in a specific food depends on the food’s nutritional and physicochemical characteristics, as well as on the temperature and the humidity of the environment [10]. The diversity of LAB and other beneficial food microorganisms, such as yeast, propionic acid bacteria, etc., is used in the food industry to create a well-tailored consortia of microorganisms that are artificially added to food to improve its quality, taste, smell, and rheological characteristics or to increase its shelf life [9]. 

During the process of yeast fermentation (alcoholic beverages, bread, and savory food), amino acids are formed through two different processes, through the decomposition of proteins by the enzymes produced by the proteolytic strains during fermentation through the Ehrlich pathway and through the activation of cereal proteases. Leucin, isoleucine, and valine, as branched-chain amino acids, can be transformed into aldehydes, which produce malty flavors, alcohols with fruity flavors, and acids with sweet, sour, rancid, rotten, fruity, and buttery flavors. The amino acids which include an aromatic ring (tyrosine, tryptophane, and phenylalanine) and sulfur-containing amino acids (methionine and cysteine) are responsible for the rose, flower, and bitter almond tastes in fermented food. Aminotransferases of lactic acid bacteria, especially *Lactobacillus* strains, produce the glutamic acid responsible for savory tastes [11].

The phenolic compounds that most widely occur in plants include phenolic acids, flavonoids, coumarins, stilbenes, tannins, lignans, and lignins. In particular, phenolic acids are found in all food groups and they are abundant in cereals, legumes, oilseeds, fruits, vegetables, beverages, and herbs in free, soluble conjugated, and insoluble bound forms, and they are mainly located in the bran and germ fractions. Although they are synthesized as first-line-of-defense chemical compounds against biotic (infections by yeasts and molds) and abiotic (nutrient deficiencies, excess cold and high UV index) stresses of plants. Phenolic acids such cafeic, ferulic, p-coumaric, and sinapic acid usually decrease during the process of fermentation. Phenolic acids, such as fumarate, lactate, succinate, and malate, are very potent natural antioxidants with proved anti-ulcer, antidiabetic, anticancer, anti-inflammatory, antiaging, antimicrobial, cardioprotective, hepatoprotective, and neuroprotective activities. Some hydroxycinnamic acids, such as ferulic acid, are especially quickly absorbed from the gastrointestinal tract, while the largest amount of phenolic compounds are esters, ethers, and amides with arabinoxylans, and they form a lignocellulose matrix resistant to digestion [12]. Generally, stilbenes are known as phytoalexins, which can be biosynthesized from grapevines as a defense to fungal diseases such as *Botrytis cinerea* or to abiotic stress and UV irradiation.

The antioxidant and antimicrobial efficiency of resveratrol provides health benefits such as the prevention of cardiovascular diseases, arteriosclerosis, and cancer. Originally, epidemiological studies indicated an inverse relationship between moderate wine consumption and the risk of coronary heart disease, the so-called ‘‘French Paradox’’ [13]. Resveratrol (3,5,40-trihydroxy-trans-stilbene) is one of the most widely studied stilbenes due to its positive impact on human health. Resveratrol, in the form of aglicon and its glcosidic analog piceid, was first discovered in the roots of the white hellebore (*Veratrum grandiflorum* Loes. fil.) but, nowadays, it is mostly recognized as the phytoalexin present in red wine [14]. 

The method used for the isolation and identification of the microbial species and the method used for the identification of metabolites synthesized by certain microorganisms present in food may greatly influence our perception of their presence in food. Regarding either beneficial or spoilage microorganisms, cognitions about the diversity of food microorganisms and their metabolites depend on the development of scientific analytical methods for their isolation and characterization. Many of them remain unknown because of the limitations of the study methods in use. The new generation of advanced molecular tools and methods offers a better understanding of microbial ecosystems and communities compared to culture isolation methods which can hamper the growth of certain microorganisms. This Special Issue of *Microorganisms* collects research and review articles on the diversity of microbial species and their metabolites with the potential to be used in tailored, innovative fermentations and other technological processes to improve the properties of food regarding its benefits for human health, paying particular attention to the employed methodologies. In addition to this, further research is needed on the synergistic effect between different microorganism strains and their impact on different food components.
